# Oxford knee score 1 year after TKR for osteoarthritis with reference to a normative population: What can patients expect?

**DOI:** 10.1016/j.ocarto.2021.100143

**Published:** 2021-02-22

**Authors:** Y.Y.W. Yap, K.L. Edwards, H. Soutakbar, G.S. Fernandes, B.E. Scammell

**Affiliations:** a School of Medicine, Queens Medical Centre, University of Nottingham, Nottingham, NG7 2UH, UK; b Centre for Sport Exercise and Osteoarthritis Research Versus Arthritis, Queens Medical Centre, University of Nottingham, Nottingham, NG7 2UH, UK

**Keywords:** Total knee replacement, Patient reported outcome measures, Oxford knee score, Normative, Patient expectations

## Abstract

**Objectives:**

Total knee replacement (TKR) procedure is commonly carried out in patients with advanced osteoarthritis to reduce pain and increase mobility, with on average 84% rated satisfactory outcome, but some (some suggest 44%) continue to experience debilitating pain. The study aimed to investigate factors affecting pain and function outcomes (using Oxford Knee Score, OKS) one year after TKR, with normative comparison to a reference population.

**Design:**

We recruited TKR patients from one hospital (Nottinghamshire, UK); collected pre- and post-operative OKS; graded radiographs for severity of osteoarthritis (K-L grade) in a sub-group. We also collected OKS by postal survey from the local area, calculated age and sex specific normative scores and z-scores of post-operative OKS (Z-OKS). The associations between K-L grade, pre-operative OKS, age, sex, against change in OKS and Z-OKS were analysed.

**Results:**

There were 536 TKR cases, 91 in radiographic sub-group and 360 people in reference cohort. Post-operative Z-OKS was associated with K-L grade (β ​= ​0.368; p<0.001). Change in OKS was associated with K-L grade (β ​= ​0.247; p ​= ​0.003); pre-operative OKS (β ​= ​−0.449; p<0.001); age (β ​= ​0.276; p ​= ​0.001); and female sex protective (β ​= ​−0.213; p ​= ​0.008). On average TKR patients returned to 74% of their normative age and sex adjusted OKS, with younger women achieving worst outcomes. More severe radiographic osteoarthritis predicted greater improvement and better post-operative outcome when compared to normative population.

**Conclusion:**

This study identified factors and provided normative OKS data intended to guide clinicians in counselling patients regarding likely surgical outcomes. This could help manage patients’ expectations, aid decision making and increase post-surgery satisfaction rate.

## 1 Introduction

Knee osteoarthritis is one of the leading causes of pain and disability worldwide [1]. Total knee replacement (TKR) is a cost-effective treatment that improves pain and function in most patients with osteoarthritis [2,3]. The procedure is indicated when initial management through education, exercise, weight loss and analgesics failed to control symptoms. However, studies estimated that up to 44% continued to experience disabling pain after surgery [4,5]. The National Joint Registry suggested that 84% of patients were satisfied with their TKR, indicating at least ‘good’ for their satisfaction level [6].

Pre-operative expectations heavily influence satisfaction level [7]. In the ranking of patients’ expectations of TKR, relieving pain and improving walking ability have been rated to be the most important [8]. As increasing prevalence of knee osteoarthritis creates greater demand for TKR in the future, it is crucial to identify factors that may predict post-operative pain and function outcomes [9]. These could be discussed with patients and aid decision making during pre-operative counselling and to manage patient expectations about what ‘success’ would look (feel) like.

The overall aim of this study was to investigate factors that affect pain and function outcomes, measured by Oxford Knee Score (OKS), 1 year after TKR for osteoarthritis, and to compare to a reference population. To achieve this, there are three objectives. 1. Identifying factors that are associated with OKS outcomes 12-months after TKR. 2. Comparing 12-month post-operative OKS with that of a population in the same county; this comparison is based on OKS, which measures self-rated pain level and ability to carry out tasks specific to the knee. 3. Identifying which factors are associated with a worse, similar or better outcome post-operatively as compared to the reference population. From a myriad of potential predicting factors, this study focuses on radiographic grading of osteoarthritis, pre-operative OKS score, age and sex.

## 2 Methods

This cohort study analyses outcomes of TKR patients at a single site in the UK (Nottingham City Hospital) with a reference population from the local (Nottinghamshire) area.

The TKR study was performed as a service evaluation of anonymised data without the need for formal ethical approval (approved by the Nottingham University Hospital NHS Trust). Ethical approval was obtained from the University of Nottingham Faculty of Medicine and Health Sciences Research Ethics Committee for the reference cohort study.

### 2.1 TKR cohort

In the TKR cohort, patients whose indication for surgery was osteoarthritis were included consecutively (2008–2010). For patients who had unilateral TKR performed on both knees within the time period, one knee was randomly selected for data analysis. These data were collected prospectively, including sex, height, weight, date of birth, date of operation, side of surgery, primary diagnosis, comorbidities, type of operation, pre-operative OKS questionnaire and the American Society of Anaesthesiologist (ASA) score, with a follow-up OKS questionnaire 12 months after operation.

Radiographic sub-group.

For radiographic assessment, a sub-group of the TKR cohort was selected, comparing patients who did not improve/improve significantly post-operatively. This is based on change in OKS (pre-operative to 12-months post-operative). Given that the minimal clinically important difference (MCID) for OKS is 5 points [10], those who had ​< ​MCID positive change in OKS were categorised as ‘Non-Improvers’ (n ​= ​98). Those with positive change in OKS of ≥25 points were categorised as ‘Top Improvers’ (n ​= ​99). 50 people were randomly selected from each group for radiograph assessment.

The Kellgren and Lawrence (K-L) system was used to evaluate severity of radiographic osteoarthritis, being one of the most commonly used [11], ranging from 0 (no OA) to 4 (severe). Each radiograph was compared against a standard radiographic atlas. Grading was based on the presence of osteophytes and joint space narrowing. Anterior-posterior, weight-bearing radiographs, taken within 6-months prior to TKR, were used to assess the tibio-femoral joint. Radiographs were assessed by a single observer (GSF) blinded to outcome scores. Inter-observer error was assessed by a second observer (BES) assessing 40/100 randomly selected films (20 per group), blinded to initial scores. Differences were assessed using the kappa statistic.

### 2.2 Reference cohort

For the reference cohort, data were collected from participants by postal questionnaire survey. From a total of 25,695 postcodes for those living in Nottinghamshire and the City of Nottingham, 2500 postcodes were randomly selected and a third allocated to each of the 3 available age categories (18–44, 45–69 and ​≥ ​70 years). For each postcode a name and address that met the required age group was randomly selected to receive the survey. The self-reported survey included age, sex, height, weight, co-morbidities and OKS questionnaire. From those who responded to the postal questionnaire (between September 2014 and April 2015), only those aged ≥30 years were included to match demographics of the TKR cohort.

All randomisation was undertaken using a random number generator by a blinded statistician.

### 2.3 Outcome data

Oxford Knee Score (OKS), the outcome measure, measures pain and function (activities of daily living) of the knee, reported by patients. This questionnaire is routinely given to patients before and after TKR [10,12]. The OKS consists of 12 questions, each rated at five levels, ranging from 0 (severe) to 4 (none). The scores were totalled to give an overall score, where 0 is the worst possible score and 48 is the best possible score. The Patient Reported Outcome Measures (PROMs) programme guideline was followed regarding missing responses [13]. Questionnaires considered as complete when there were a maximum of 2 missing responses; mean of remaining responses imputed for missing values.

There were two outcome variables: change in OKS (pre-operative to post-operative) and 12-month post-operative standardised Z-score of OKS (Z-OKS). The former shows the 12-month change since TKR and the latter indicates how the post-operative OKS compares to age and sex matched reference population.

### 2.4 Data analysis

All three cohort demographics were described: providing mean/SD for the continuous variables (age, BMI); median/inter-quartile range (IQR) for OKS (pre-operative/post-operative); and count/percentages for categorised data (sex, ASA, osteoarthritis status).

For the first objective, the change in OKS between the two time points for the TKR cohort were described (median/IQR) as well as categorised by age (<60 years, 60–69years, ≥70years), sex (male/female), and K-L grade (0–1, 2 and 3–4). OKS at the two time points were compared using Mann-Whitney U test. Next, multivariate regression analyses were undertaken: change in OKS as outcome variable; independent variables were age, sex, pre-operative OKS and K-L grade. The model was run twice, with and without K-L data (as only the sub-group had these data).

For the second objective, analyses were made with reference to an age and sex matched reference cohort. First, the post-operative OKS data were described: categorised by age, sex, and pre-operative OKS category (dichotomised, high/low, around its median value). Then the post-operative OKS was compared to OKS of the age and sex matched reference population (post-operative OKS of TKR cohort divided by OKS of matched reference population, expressed as a percentage). Next, the post-operative OKS was used to calculate individual post-operative Z-OKS. The reference cohort were split into six groups by sex and age (male/female; <60,60–69,≥70 years) and mean and standard deviations of their OKS calculated. Z-OKS was the difference between TKR cohort post-operative OKS value and reference cohort OKS mean, divided by the standard deviation of the reference cohort (age and sex stratified). A negative z-score indicated the patient was below the equivalent figure for the age and sex matched reference group and vice versa. The proportion of TKR cohort with a positive Z-OKS were calculated.

For the third objective, multivariable regression analysis was conducted with post-operative Z-OKS as outcome variable; K-L grade and pre-operative OKS as independent variables. Clearly, age and sex could not be included in this model. This analyse was to investigate how pre-operative OKS and K-L grade predict better/worse outcomes in men/women of different ages.

Statistical analyses were conducted using SPSS (version 22.0, IBM). A p-value <0.05 was considered significant.

## 3 Results

TKR study cohort**:** Data from a total of 869 TKR patients were obtained during the study period. 605 (69.6%) patients had completed questionnaires at both time points. After exclusion (37 had TKR for indications other than osteoarthritis; 32 had bilateral knee TKR), 536 (88.6%) patient knees were analysed. The cohort characteristics are presented in Table 1. The patients’ age ranged from 37 to 92 years (mean 70 years). There was a higher representation of females (60%) and obese patients (59%). According to the ASA score, most were classified as having mild systemic disease. All patients underwent a cemented TKR. The median OKS improved 17.6 points between pre- and post-operative (Table 2). Patients were dichotomised as ‘low’ pre-operative OKS if the score was less than the median value (<17 points) (n ​= ​269), or ‘high’ score if it was ≥17 points (n ​= ​267).

**Table 1:** Patient characteristics of the TKR and reference cohorts.

Variable	TKR Cohort (n ​= ​536)		Radiographic sub-group (n ​= ​91)		Reference Cohort (n ​= ​360)	
	n (%)	Mean ​± ​SD	n (%)	Mean ​± ​SD	n (%)	Mean ​± ​SD
**Age (years)**		70.0 ​± ​9.5		69.8 ​± ​9.0		65.1 ​± ​13.6
**BMI (kg/m**^**2**^**)**		31.7 ​± ​5.7 (n ​= ​421)		33.2 ​± ​10.1 (n ​= ​69)		26.7 ​± ​5.2 (n ​= ​346)
**ASA (1)**	29 (5%)		6 (7%)		n/a	
**ASA (2)**	411 (78%)		63 (72%)		n/a	
**ASA (3)**	83 (16%)		17 (20%)		n/a	
**ASA (4)**	4 (1%)		1 (1%)		n/a	
**Female**	321 (60%)		69 (76%)		198 (55%)	
**Male**	215 (40%)		22 (24%)		162 (45%)

**Table 2:** Patient OKS for the TKR and reference cohorts.

Variable	TKR Cohort (n ​= ​536)	Radiographic sub-group (n ​= ​91)	Reference Cohort (n ​= ​360)	
	Median (IQR)	Median (IQR)	n	Median (IQR)
Pre-operative OKS	16.4 (12.0–22.0)	14.0 (10.0–19.5)		46.0 (37.0–48.0)
Post-operative OKS	34.0 (24.0–40.8)	34.0 (34.0–41.0)		n/a
Without osteoarthritis			268	47.0 (41.8–48.0)
With osteoarthritis			92	38.0 (29.0–43.0)
With osteoarthritis, no TKR			70	39.0 (33.0–44.0)
With osteoarthritis, had TKR			22	29.8 (24.3–39.8)

Radiographic sub-group**:** Out of the 100 patients randomly selected from the ‘Non-improver’ and ‘Top-improver’ groups, 8 were excluded due to radiographs being non weight-bearing or taken more than six months before surgery, 1 was excluded as radiograph showed signs of rheumatoid arthritis. Thus 91 radiographs were assessed. These patients were similar to the TKR cohort in terms of age, BMI and ASA, with slightly more females (76%) (Table 1). There were 55 (60%) patients in the low pre-operative OKS group and 36 (40%) in the high group. The intra-observer reliability scores showed moderate reproducibility for K-L grade (Kappa value of 0.750). The inter-observer reliability scores showed high reproducibility (Kappa value of 0.722).

Reference cohort: 409 returned questionnaires (response rate 16.4%), of which 362 (88.5%) were complete. After exclusion (2 were <30 years old), 360 (99.4%) questionnaires were used in analysis. The ages ranged from 33 to 90 years (mean 65 years), 55% female, and mean BMI 26.7 ​kg/m^2^ (Table 1). 92 (26%) of the reference cohort had osteoarthritis, of which 22 had had a TKR. For the whole cohort, the OKS ranged from 3 to 48, with a median of 46 (IQR: 37–48). Those who had osteoarthritis reported lower median OKS (38.0) than those without (47.0) (p<0.001). Of those with osteoarthritis, respondents after TKR reported lower median OKS (29.8) than those without TKR (39.0) (p<0.001).

### 3.1 Change in OKS

Table 2 shows the change in median OKS from 16.4 to 34.0 between pre-operative and post-operative time points for the TKR cohort, which was statistically significant (95% CI 14.0, 15.7; p<0.001). The change in OKS data were negatively skewed, indicating that most patients experienced improvement after the operation. 40 patients (7.5%) reported lower (worse) OKS post-operatively (negative change in OKS). 58 patients (10.8%) achieved no clinical improvement (change in OKS between 0 and 5).

These data were then categorised by age, sex and K-L grade (Table 3). The median and interquartile range (IQR) data are presented for pre-operative and post-operative OKS as well as the change between the two scores in Table 3, with totals for the TKR group. This shows that whilst all three age groups had similar pre-operative median OKS, the younger age group performed worst on average (lowest scores) post-operatively, with the lowest change in OKS (11 points). Females’ median pre-operative OKS were lower than males’ (16 vs 18 respectively), but a slightly higher change in OKS (17.0 vs 14.3 respectively) resulted in similar median post-operative OKS. Patients in the lowest K-L grade group, had higher median pre-operative OKS and lower median post-operative OKS. This group had a negative change in OKS, which was also the lowest median change (worse than before). There were small numbers in this group (n ​= ​9).

**Table 3:** Median pre-operative, post-operative and change in OKS by age, sex and K-L score for TKR cohort (n ​= ​536) and the radiographic sub-cohort (n ​= ​91).

Variables		N	Median (IQR) Pre-operative OKS	Post-operative OKS	Change in OKS
**K-L grade**	0–1	9	22.0 (10.0–27.0)	20.0 (12.0–24.0)	−2.0 (−3.0–2.0)
	2	19	15.0 (13.1–22.0)	22.0 (16.0–40.0)	4.0 (−4.0–26.0)
	3–4	62	13.0 (8.0–17.0)	37.0 (21.0–42.0)	26.0 (3.0–29.0)
**Age**	<60	79	16.0 (12.0–21.0)	27.0 (18.0–37.0)	11.0 (2.0–19.0)
	60–69	166	17.0 (13.0–22.0)	35.0 (23.0–41.0)	15.5 (7.0–23.0)
	≥70	291	17.0 (12.0–22.0)	34.0 (27.3–40.0)	16.0 (10.0–23.0)
**Sex**	Female	321	16.0 (11.0–20.0)	34.0 (25.0–40.0)	17.0 (8.0–24.0)
	Male	215	18.0 (14.0–23.0)	34.0 (24.0–41.0)	14.3 (7.0–20.0)
**Total n536**		536	16.4 (12.0–22.0)	34.0 (24.0–40.8)	15.0 (8.0–22.0)

When these data were analysed in a multivariate regression model (outcome variable: change in OKS) with age, sex and pre-operative OKS, as independent variables (n ​= ​536). This model explained 11.8% (11.4% adjusted) of the variation in the change in OKS (p<0.001). K-L grade was then added to the model (n ​= ​91); it explained 49.8% (47.4% adjusted) of the outcome variation (p<0.001) (Table 4). All four variables showed a statistically significant relationship with change in OKS (Table 4). Pre-operative OKS showed the biggest adjusted effect size (p<0.001) with lower pre-operative OKS associated with higher change in OKS (improvement). Sex also showed a negative relationship, with women showing a greater change in OKS (p ​= ​0.008). Sex was not statistically significant in the initial model but was in the radiographic sub-group analyses. Age and K-L grade both reported positive associations, so younger patients and those with lower K-L grade showed the least improvement in OKS, and vice versa (p ​= ​0.001 and p ​= ​0.003, respectively). However, the difference in median change in OKS between the three age categories was marginally less than the MCID of 5 points (4.5 points between <60 and 60–70 years; 5.0 points between <60 and >70 years).

**Table 4:** Summary of multivariate regression model results for change in OKS for the TRK cohort (n ​= ​536) and for radiographic sub-group (n ​= ​91).

	Change in OKS for TKR cohort (n ​= ​536)		
	β (95% CI)	Adjusted β	p value
**Pre-operative OKS**	−0.448 (−0.568, −0.329)	−0.306	0.001
**Sex (0 female; 1 male)**	−0.926 (−2.616, 0.764)	−0.045	0.282
**Age**	0.152 (0.067, 0.238)	0.143	<0.001
	**Change in OKS for radiographic sub-group (n=91)**		
	**β (95% CI)**	**Adjusted β**	**p value**
**Pre-operative OKS**	−0.908 (−1.231, −0.585)	−0.449	<0.001
**Sex (0 female; 1 male)**	−7.381 (−12.797, −1.965)	−0.213	0.008
**Age**	0.459 (0.202, 0.716)	0.276	0.001
**K-L category**	5.608 (2.027, 9.188)	0.247	0.003

### 3.2 Normative data

The second objective is examined next, exploring how patients fared compared to a reference population.

Table 5 shows the median postoperative OKS of the TKR group and the current OKS for the reference cohort, categorised by sex, age (both groups) and pre-operative OKS (TKR group only) categories. On average, TKR patients did not return to the same levels as the Reference group post-operatively, achieving 74% of the Reference group OKS, with women <60 years ‘performing’ the least well (55% of the score in a ‘normative’ population: OKS 26 vs 47 points respectively). Table 5 also highlights that median post-operative OKS was lower for patients with low pre-operative OKS across all age and sex groups. This difference was greatest for young (<60 years) males (23 points for low pre-operative OKS and 41 points for high pre-operative OKS).

**Table 5:** Median post-operative OKS of TKR and current OKS of Reference cohorts, categorised by sex and age for both groups and further categorised by pre-operative OKS for the TKR cohort.

Sex	Age group	Pre-operative OKS group	n	TKR cohort Post-operative OKS	Post-operative OKS as % of reference cohort	n	Reference cohort current OKS
				Median (IQR)	%		Median (IQR)
Female	<60	Low	25	24.0 (14.0–31.0)			
		High	24	29.0 (20.5–38.0)			
		All	49	26.0 (18.0–36.0)	55.3	65	47.0 (45.0–48.0)
	60–69	Low	56	32.5 (21.0–41.0)			
		High	47	37.0 (32.0–43.0)			
		All	103	36.0 (26.0–42.0)	80.0	65	45.0 (37.0–48.0)
	≥70	Low	101	32.0 (24.0–38.0)			
		High	68	38.0 (30.0–42.6)			
		All	169	34.0 (27.0–39.0)	79.4	68	42.8 (30.0–47.0)
		All females	321	34.0 (25.0–40.0)	73.9	198	46.0 (38.2–48.0)
Male	<60	Low	17	23.0 (17.0–27.0)			
		High	13	41.0 (38.0–44.0)			
		All	30	32.0 (20.2–40.0)	71.1	28	45.0 (36.0–47.0)
	60–69	Low	26	23.0 (19.0–36.0)			
		High	37	36.0 (26.0–42.0)			
		All	63	36.0 (26.0–42.0)	78.3	75	46.0 (40.0–48.0)
	≥70	Low	44	30.8 (22.5–37.0)			
		High	78	37.0 (33.0–43.0)			
		All	122	35.0 (28.2–42.0)	83.3	59	42.0 (27.0–48.0)
		All males	215	34.0 (24.0–41.0)	74.7	162	45.5 (34.2–48.0)
Total		All M&F	536	34.0 (24.0–40.2)	73.9	360	46.0 (37.0–48.0)

The Reference Cohort data were used to calculate the Z-OKS (Table 6). The Z-OKS ranged from −7.586 to 0.899, with mean of −1.0 (SD 1.57). By sex, the Z-OKS ranged from −7.586 to 0.872 for females (mean −1.0; SD 1.61) and −5.553 to 0.899 for males (mean −1.1; SD 1.52) (a negative z-OKS indicates the patient fared less well than that of the reference cohort) (Table 6). That is, on average females fared less well than males. By age and sex, females aged less than 60 years had the lowest positive results (0.377 compared to the highest rated value of 0.872); while males in the age group of 60–69 had the lowest positive result (0.619 compared to the highest rate value of 0.899). Generally, older patients were more likely to have a better (higher) OKS than the Reference group. Overall, 27% of participants had a Z-OKS>0 (i.e. better than the Reference group). That is, over 70% of patients did not return to an age and sex matched ‘norm’ post-operatively.

**Table 6:** Mean (SD) post-operative OKS of TKR and current OKS of Reference cohorts together with the z-OKS ranges, categorised by sex and age for both groups.

Sex	Age group	TKR cohort			Mean OKS as % of reference cohort	Reference cohort			Z-OKS mean (SD), range for TKR cohort	Positive Z-OKS n (%)
		n	%	Post-operative OKS, Mean (SD)		n	%	Current OKS Mean (SD)		
Female	<60	49	15.3	26.4 (11.42)	58.7	65	32.8	45.0 (5.40)	−3.4 (2.14), −7.586 to 0.377	1 (2)
	60–69	103	32.1	33.2 (11.18)	80.4	65	32.8	41.3 (9.07)	−0.9 (1.23), −3.783 to 0.736	27 (26)
	≥70	169	52.6	32.6 (9.45)	87.2	68	34.3	37.5 (12.11)	−0.4 (0.78), −2.762 to 0.872	64 (38)
	All female	321	59.9	31.9 (10.57)	77.4	198	55.0	41.2 (9.82)	−1.0 (1.61), −7.586 to 0.872	92 (29)
Male	<60	30	14.0	29.8 (12.33)	75.4	30	18.5	39.6 (9.38)	−1.0 (1.32), −3.793 to 0.899	9 (30)
	60–69	63	29.3	30.0 (10.88)	67.7	73	45.1	44.3 (6.00)	−2.4 (1.82), −5.553 to 0.619	4 (6)
	≥70	122	56.7	34.3 (8.78)	88.8	59	36.4	38.7 (11.10)	−0.4 (0.79), −2.494 to 0.841	41 (34)
	All male	215	40.1	32.4 (10.16)	80.3	162	45.0	40.4 (9.93)	−1.1 (1.52), −5.553 to 0.899	54 (25)
Total		536		32.1 (10.4)	78.6	360		40.8 (9.88)	−1.0 (1.57), −7.586 to 0.899	146 (27)

Finally, the third sub-objective is examined. Regression models with Z-OKS as the outcome variable are presented in Table 7, the former for the TKR cohort (n ​= ​536), the latter for the radiographic sub-group (n ​= ​91). The first model with pre-operative OKS explained 7.1% (adjusted 7.0%) of the variation in the Z-OKS (p<0.001). The coefficient of determination doubled to 14.2% (adjusted 12.3%) when K-L grade was added to the model (p ​= ​0.001). K-L grade had a positive association with Z-OKS (standardised coefficient ​= ​0.368, p<0.001). This means a higher K-L grade pre-operatively was associated with a higher Z-OKS (that is, more likely to have a higher post-operative OKS than that of the reference cohort data) after TKR. Pre-operative OKS had a weak negative association (standardised coefficient ​= ​−0.037, p ​= ​0.715).

**Table 7:** Summary of regression model results for post-operative Z-OKS for TKR cohort (n ​= ​536) and radiographic sub-group (n ​= ​91).

	Post-Operative Z-OKS for TKR cohort (n ​= ​536)		
	β (95% CI)	Adjusted β	p value
**Pre-operative OKS**	0.060 (0.042, 0.079)	0.267	<0.001
	**Post-Operative Z-OKS for radiographic sub-group (n=91)**		
	**β (95% CI)**	**Adjusted β**	**p value**
**K-L grade**	1.139 (0.518, 1.759)	0.368	<0.001
**Pre-operative OKS**	−0.10 (−0.066, 0.045)	−0.037	0.715

## 4 Discussion

In this study, majority of the patients experienced improvement in OKS one year after TKR, with the median post-operative OKS similar to that in other studies [[14], [15], [16], [17]]. On average, TKR patients in our study achieve 74% of their normative age and sex adjusted OKS levels after surgery, with younger patients (<60 years), particularly women, achieving the worst outcomes. Pre-operative K-L grade is important in determining outcome, with patients with less severe scores achieving the least improvement.

### 4.1 Applications to clinical practice

The normative values in Table 5, Table 6 could aid patients form realistic expectations of pain and functional (OKS) outcomes one year after TKR. Some patients experience mild pain and little functional limitations after surgery. However, on average, patients could expect moderate pain and functional limitations. For instance, they could experience moderate pain when standing up from a chair and have moderate difficulty kneeling and getting up again. Overall, one year after having TKR, their OKS level could improve to just over 70% of that experienced by others of similar age and sex.

### 4.2 Radiographic findings

This analysis suggested that having more severe osteoarthritis on radiographs was associated with greater improvement and better post-operative OKS outcomes. This positive association is consistent with several studies [[18], [19], [20]]. In Valdes et al., the study (n ​= ​869) used only post-operative WOMAC scores to assess pain and it concluded that those with more severe K-L grade had better post-operative pain outcomes [18]. However, as pre-operative pain scores were not obtained, the degree of improvement was not compared. In another study (n ​= ​478) that included pre-operative scores and used IKSS in its analysis, Dowsey et al. found that those with less severe radiographic changes are less likely to experience major improvement in pain and function 1 and 2 years after operation [19], which concurs with this work. These findings support the use of TKR for patients with more advanced osteoarthritis.

Some studies have shown that local and systemic inflammation is correlated with pain [21]. Moreover, in Lundblad’s study comparing rest pain and pain on movement, it was noted that the association between high radiographic osteoarthritis and better post-operative pain outcome only applies to pain related to movement [20]. This pain is more closely related to pathology in knee joint which is mitigated by surgery.

However, some studies have found that although those with more severe radiographic changes may experience major improvements, they do not attain the same level of post-operative function as those with less severe changes [5,22]. This study did not fully concur. This study measured post-operative function in two ways, both the absolute post-operative OKS score and the Z-OKS, determining how the patient’s OKS compares to a sex and age matched normative population. We found a positive association between K-L grade with both change in OKS and the z-score, thus demonstrating that patients with more severe pre-operative radiographical changes experienced more change in OKS (improvement) (p ​= ​0.003) and also fared better after TKR compared to the Reference group than patients with less severe pre-operative radiographical changes (p<0.001). That is, 25–30% of patients (depending on sex and age, with females and older patients faring better) had a positive Z-OKS, suggesting their score was higher than for the age and sex matched reference population. This suggests that patients with more severe radiographic findings were more likely to do well post-operatively.

The discrepancy between radiological findings and pain symptoms is widely known [1,23]. A low K-L grade can be associated with high pain; this pain is likely due to a different mechanism than for those patients with high K-L grade and unlikely to be comprehensively addressed by surgery. Pain is influenced by many other factors, including psychosocial factors and central sensitisation, which may not be resolved as easily. In severe osteoarthritis, patient might have progressed to have central pain sensitisation and functional decline, which would reduce the surgical improvement [[18], [19], [20]].

### 4.3 Pre-operative OKS finding

Another key finding is that although lower pre-operative score is associated with greater improvement in OKS, the post-operative outcome is still worse than patients with higher pre-operative score. This is consistent with several studies [5,14]. It is rational that patients who had more OKS impairment experienced more improvement after TKR. However, despite the greater improvement, they still experienced more pain than those who had better pre-operative pain score [5,18,20]. It is noted that it is also possible to interpret that patients with lower pre-operative OKS may have experienced a higher change in OKS (Table 4) simply because they have more ‘room’ to improve their score.

This could be explained by the multifactorial nature and subjectivity of pain. Patients themselves giving ratings before and after surgery levels on factors like their pain perception and thresholds. However, other factors like their expectations and psychosocial factors would still affect how the ratings are given [4,5]. Central sensitisation was also postulated as a key reason for the high pain levels experienced before and after surgery, as these are not fixed by the operation [5,20]. Therefore, clinicians should consider pre-operative OKS with psychosocial conditions when evaluating the benefits of TKR for each patient.

### 4.4 Strengths & limitations

This study has several strengths. Measuring outcome at 12 months after operation is a reasonable time point for assessment as studies have shown that most improvement occurs within the first year, and tends to plateau thereafter [8,24]. Also, as the reference population is from the same county, differences between comparison groups such as environmental, psychosocial factors are reduced.

Another strength of this study is using both change in score and standardised scores as outcome variables. Change in scores is a more accurate reflection of TKR outcomes as compared to those who analysed post-operative scores alone. By standardising post-operative outcomes with that of a general population, the floor and ceiling effect often observed in fixed-end scales like OKS is reduced. Analysing the outcomes using both variables simultaneously reinforced the findings.

In addition, this study uses OKS which is reliable and used in the wider PROMs programme in England and Wales. OKS can be easily completed by patients themselves, without the influence of clinicians. This removes reporting bias. The questions in OKS are also specific for the knee joint, reducing the influence of unrelated comorbidities on their rating. Nevertheless, some comorbidities such as hip pathology could still affect the individual’s ability to carry out some tasks described in the questionnaire. This study also did not explore other factors such as comorbidities, presence of complications, previous trauma and knee surgery. BMI, though collected in the study, has not been included in analysis due to high level of missing data. All of these could act as confounders.

One of the limitations in our study was the validity of reference cohort as representation of the county population. As the data obtained for reference population was based on voluntary participation, it is subjected to response bias.

Secondly, it should be noted that most patients in this study had high K-L grade (3 or 4), with only 9 having low K-L grade (0 or 1). This is understandable as TKR is usually performed when symptoms of osteoarthritis are refractory to medical management. Also, in this study, only anterior-posterior radiographs were used to assess tibiofemoral K-L grade of osteoarthritis. Fewer patients had skyline radiographs taken, which could have shown patellofemoral osteoarthritis.

Lastly, our analysis is based on a single site, which may limit the generalisability of study findings. All study subjects were from one hospital, which can be subjected to selection bias. Yet, the patient demographics in this study were similar to that reported by National Joint Registry for TKR performed in 2010 in terms of age, proportion of sex and ASA score [25]. As data for the TKR cohort was prospectively collected and around one third of participants were lost over follow up period, non-response bias was present.

## 5 Conclusion

A novel finding from this study is the comparison of outcomes in those who had TKR with the general population in the same county. The data stratified by age and sex could help patients formulate post-operative expectations more easily. The mismatch between expectations and outcomes has been known as one of the factors for poor satisfaction, thus this information could help increase satisfaction rate after procedure [7].

This study could help guide clinicians in counselling patients on management options for osteoarthritis, including TKR procedure. In addition to factors analysed in this study, various factors need to be considered collectively for holistic management of each individual. If patients who are less likely to benefit from TKR are identified, different treatment options could be fully explored. By providing information to help patients form more realistic expectations of outcomes after surgery, patients could give more informed consent, and potentially increase their satisfaction rate.

## Author contributions

YY: Contributed to the study design, literature review, statistical analysis and writing of manuscript, responding to reviewers, reviewing final drafts. GF: Contributed to the grading of radiographs and drafting of manuscript, reviewing final drafts. HS: Contributed to data collection, analysis of the reference cohort and drafting of manuscript, reviewing final drafts. BS: Contributed to conception and design of study, grading of radiographs (for inter-observer error check), data interpretation and drafting of manuscript, responding to reviewers, reviewing final drafts. KE: Contributed to the study design and statistical analysis, interpretation and writing of manuscript, responding to reviewers, reviewing final drafts.

## Role of funding source

This study was funded by the 10.13039/501100000837University of Nottingham and by the Centre for Sport, Exercise and Osteoarthritis Research 10.13039/501100012041Versus Arthritis (Grant reference 21595).

## Declaration of competing interest

The authors have no conflicts of interest.
